# Embolization of Perforated Coronary Artery with a Fragment of Balloon Catheter (Cut Balloon Technique)—Multicenter Study

**DOI:** 10.3390/jcdd10120496

**Published:** 2023-12-14

**Authors:** Grzegorz Sobieszek, Bartosz Zięba, Wojciech Dworzański, Rafał Celiński, Umberto Barbero, Maksymilian P. Opolski

**Affiliations:** 1 Department of Cardiology, 1st Military Hospital, 20-049 Lublin, Poland; grzes.bies@interia.pl; 2 Department of Cardiology, Specialistic Hospital, 26-610 Radom, Poland; wojciech686@wp.pl; 3 Department of Cardiology, Specialistic Hospital, 22-100 Chełm, Poland; rcelinski@op.pl; 4 Cardiology Division, Santissima Annunziata Hospital, 12038 Savigliano, Italy; umberto.barbero@unito.it; 5 Department of Interventional Cardiology and Angiology, National Institute of Cardiology, 04-628 Warsaw, Poland; opolski.mp@gmail.com

**Keywords:** percutaneous coronary intervention, coronary artery disease, coronary perforation, embolization, cut balloon technique

## Abstract

Background: Iatrogenic distal coronary artery perforation can be a life-threatening complication. While there are different dedicated devices for the embolization of distal perforations, there are scarce data about the embolization using the fragmented balloon catheter, the so-called cut balloon technique (CBT). Methods: We included consecutive patients with distal coronary perforations treated with CBT in four cardiac centers between 2017 and 2023. Clinical, angiographic and procedural characteristics as well as in-hospital outcomes were recorded. Results: Twenty-six patients (68% men, mean age: 71 ± 10.6 years) with 25 distal coronary perforations and one septal collateral perforation were included. Eleven patients (42%) had elective percutaneous coronary intervention, while fifteen patients (58%) were treated for acute coronary syndrome. The site of perforation was most frequently distributed in the left anterior descending artery (40%), followed by the circumflex artery (28%) and right coronary artery (24%). The diameter of balloons for CBT ranged from 1.5 to 4.0 mm, with most balloons (76%) being either 2.0 or 2.5 mm in diameter. Most balloons (88%) were previously used for lesion predilatation. The numbers of cut balloons needed to seal the perforation were 1, 2 and ≥3 in 48%, 20% and 32% of cases, respectively. The in-hospital prognosis was favorable, with cardiac tamponade requiring pericardiocentesis in only four (16%) patients. Neither emergency surgery nor cardiac death occurred. Conclusions: CBT is a safe, efficient and easy-to-implement technique for the embolization of coronary perforations. Most distal coronary perforations can be sealed with one or two fragments of cut balloons, obviating the need for additional devices.

## 1. Introduction

Iatrogenic coronary perforation can be a life-threatening complication, resulting in cardiac tamponade and hemodynamic instability. Coronary perforations are classified according to the location (large vessel, distal vessel and collateral perforation) [1], severity and the associated risk of tamponade (Ellis classification) [2]. Distal coronary perforations are often caused by deep guidewire penetration into a small branch, and occur with a frequency of approximately 0.13–0.35% during all percutaneous interventions (accounting for 25% of all coronary perforations) [3,4]. Of particular interest, the occurrence of distal perforation was linked to the use of hydrophilic guidewires as well as the complexity of percutaneous coronary intervention (PCI), and it resulted in cardiac tamponade in almost half of the cases [3,5].

The management of distal perforations relies on the immediate hemodynamic stabilization (if necessary) and sealing of the perforation with the use of coils, thrombin, clot, fat, glue or spongostan [4,6,7,8]. Notably, while most of these specialized materials are not commonly available and require microcatheters for targeted deployment, there is an unmet need for a universal and simple treatment option for distal coronary perforation. Particularly germane to this concept, in 2020, the method for the embolization of distal coronary perforation using a fragment of a cut balloon catheter—the so-called “cut balloon technique” (CBT) or ”cut remnant of a used angioplasty balloon”—was first described [9]. The aim of the current report is to describe the multicenter experience of clinical, angiographic and procedural characteristics of distal coronary perforations treated by the CBT.

## 2. Methods

We included consecutive patients with distal coronary perforations treated with the CBT in 4 cardiac centers between 2017 and 2023. We retrospectively analyzed the baseline clinical, angiographic and procedural characteristics. The study protocol was approved by the institutional ethics committee, and informed consent was waived.

Distal coronary perforations were diagnosed by coronary angiography during PCI. The distal coronary perforation and collateral perforation were defined according to Brilakis et al.’s anatomical classification as a perforation of the distal part of the main vessel or side branch, usually caused by distal wire migration [10]. Cardiac tamponade was defined as the presence of systemic hypotension (systolic blood pressure < 90 mm Hg) along with echocardiographic features of cardiac tamponade (pericardial fluid collection, early diastolic collapse of the right ventricle and late diastolic right atrial collapse) [11]. Target coronary lesions were classified according to the ACC/AHA classification criteria [12].

**Figure 1:**
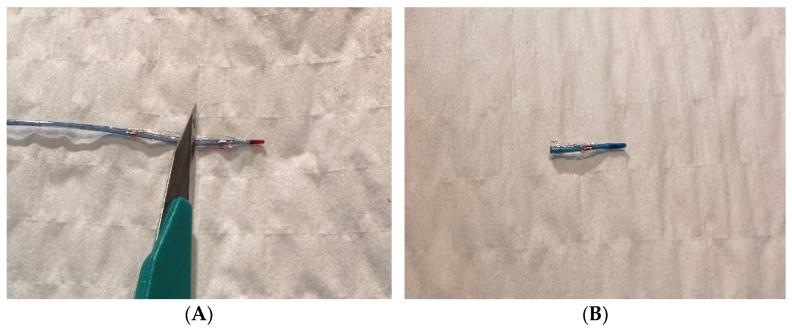

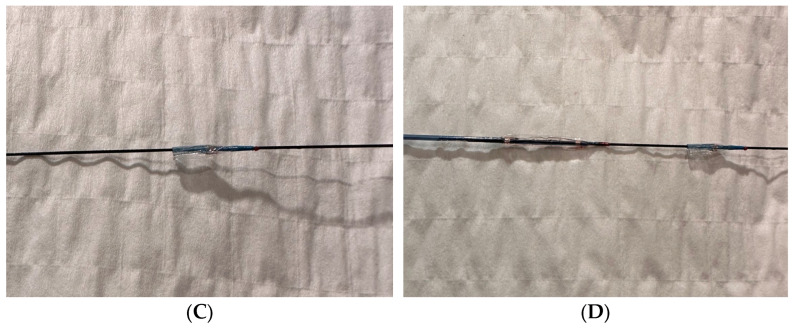
The cut balloon technique (CBT). (**A**) Semi-compliant balloon cut in the middle, (**B**) distal part of the balloon, (**C**) distal part of the balloon on the coronary wire, (**D**) balloon being pushed together with distal part of the cut balloon on the coronary wire.

**Figure 2:**
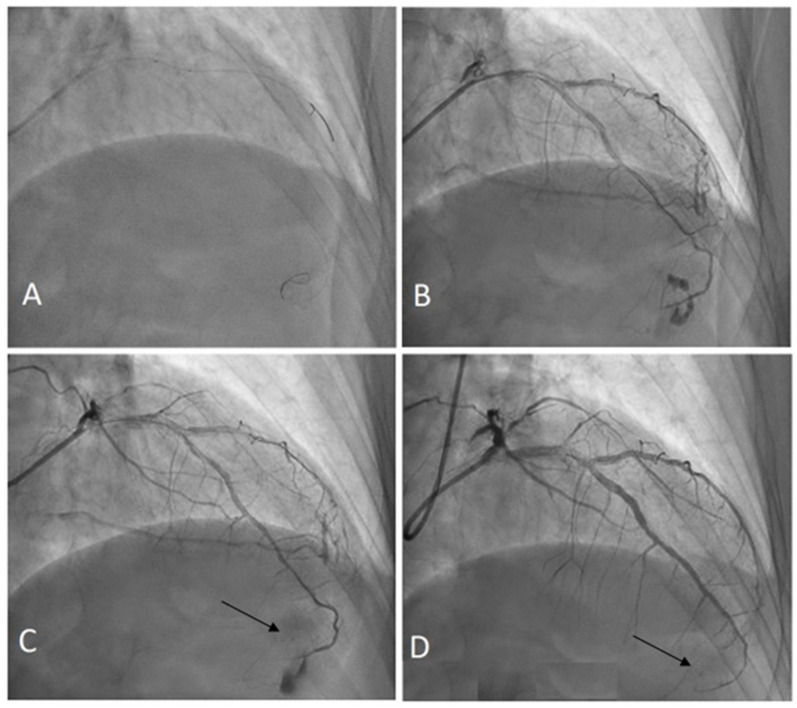
Distal LAD perforation sealed using the CBT. Arrows show the marker of the fragmented balloon. (**A**) Deep wire penetration, (**B**) distal perforation, (**C**) CBT embolization, (**D**) control angiography after 8 days.

The comprehensive step-by-step application of the CBT was described in a prior publication [9]. Essentially, it was preceded by a prolonged balloon inflation proximal to the perforation site in order to limit the coronary leakage while preparing the CBT. Time of prolonged balloon inflation was dependent on CBT application, and usually took less than 10 min. In brief, the previously expanded balloon (semi-compliant or noncompliant) should be cut between the markers. Next, the resulting umbrella-shaped cut remnant of the balloon is put on the coronary wire and placed in the distal part of the perforated vessel by pushing it forward with a second non-fragmented (new) balloon catheter [8]. The schematic illustration of CBT is shown in Figure 1 and Figure 2.

Heparin reversal with protamine was used individually and at the discretion of the operator. As a general rule, protamine was administered at the dose of 50 mg i.v. in cases associated with tamponade. During the acute phase of complication, coagulation was not assessed using the activated clotting time.

## 3. Results

The baseline characteristics, comorbidities and details of the procedure are presented in Table 1. A total of 11 out of 26 patients with coronary perforation (42%) had elective PCI, while 15 patients (58%) were treated for acute coronary syndrome. Chronic total occlusion PCI was performed in only 3 patients.

In the study population, there were 25 cases of distal coronary perforation and 1 case of septal collateral perforation among 26 patients (68% men, mean age: 71 ± 10.6 years). All distal coronary perforations were treated with the CBT alone, while the septal collateral perforation was treated with two cut balloons accompanied with one coil at the discretion of the operator. No other techniques were used to close the distal perforation in our cohort. The sizes of the balloons for the CBT ranged from 1.5 mm to 4.0 mm, with 76% of the balloons being either 2.0 mm or 2.5 mm in size. Most balloons (88%) used in the CBT were previously used for lesion predilatation. We did not use any additional techniques to deliver the distal part of the balloon (e.g., ping pong technique).

The site of distal coronary perforation was most frequently distributed in the left anterior descending coronary artery (40%), followed by the circumflex artery (28%) and the right coronary artery (24%). Of note, in the majority of cases, coronary perforation was associated with the use of non-hydrophilic guidewires (92%) and multiple (≥2) guidewires (64%). The number of cut balloons needed to seal the perforation was 1 in 48% of cases, 2 in 20% of cases and ≥3 in 32% of cases (Table 2).

In a single case of septal collateral perforation encountered during chronic total occlusion PCI, two cut balloons and one coil were implanted for the assurance of the complete embolization of the extravasation site. The in-hospital prognosis was favorable with cardiac tamponade, requiring pericardiocentesis in four (16%) patients only. Neither emergency surgery nor cardiac death occurred.

## 4. Discussion

To the best of our knowledge, this is the first multicenter study to comprehensively evaluate the feasibility and efficacy of the CBT for the treatment of coronary perforations encountered during PCI. Our results confirm the high efficacy of the CBT in the embolization of distal coronary perforations distributed across all three major coronary arteries, as reflected by the good in-hospital prognosis with low rates of cardiac tamponade requiring pericardiocentesis. Of particular interest, most perforations in our cohort (68%) could be sealed with one or two fragments of cut balloons, obviating the need for additional devices and/or sophisticated techniques. Notably, our findings may be considered fairly reproducible given the simplicity, safety and high availability of CBT across most catheterization laboratories.

The clinical and angiographic characteristics of our study are In line with prior reports on distal coronary perforations [3,13]. Specifically, most of the examined cases involved patients with acute coronary syndrome, and the most frequent site of distal perforation was in the left anterior descending coronary artery territory. In addition, the majority of our perforations occurred when more than one guidewire was used. Interestingly, and in contrast to a prior study in the literature [14], most perforations were induced by non-hydrophilic guidewires, which is an observation that might be explained by patient and/or operator selection bias.

Since its first description in 2020, there have been very limited data (restricted to case reports and single-center case series) on the use of the CBT in clinical practice [15,16,17]. In this regard, our report is the first multicenter study to confirm the feasibility of CBT for the treatment of distal coronary perforations, adding yet another technique to the PCI armamentarium. Of particular interest, our results corroborate the good safety profile of the CBT. Indeed, the use of the CBT resulted in the complete embolization of all attempted distal perforations without further clinical sequelae. Specifically, only 4 out of 25 patients (16%) developed tamponade, requiring pericardiocentesis. Moreover, neither emergency surgery nor cardiac death were reported following the use of the CBT.

In our opinion, the CBT has several important advantages compared to other embolization techniques. Practically, it is simple, reproducible, safe, cheap and commonly available in most catheterization laboratories (Table 3). From the technical point of view, the cut balloon is pushed over the coronary guidewire to the site of distal perforation by a second new and non-fragmented balloon. We assume that the mechanism of the CBT in treating distal coronary perforation is the same as in other methods (coils, fat and clot) and relies upon stopping the blood flow and initiating local blood clot formation. Importantly, because of the presence of a radiopaque marker on the cut balloon, its position in the target vessel can easily be determined.

**Figure 3:**
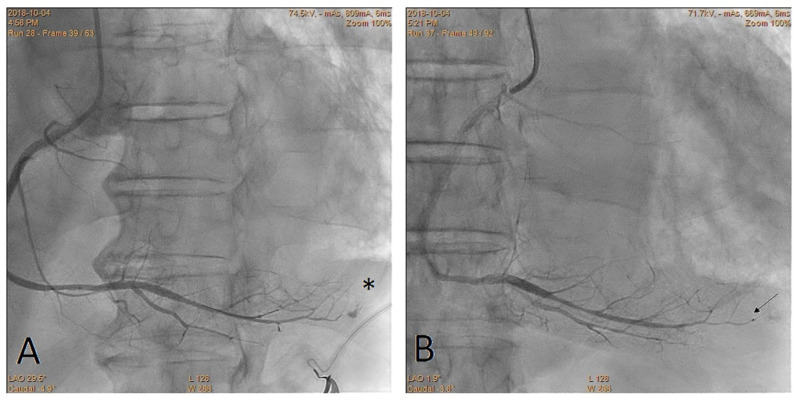
Perforation of the distal right coronary artery. The star shows the location of the perforation. The arrow shows the marker of the fragmented balloon. (**A**) Before the CTB; (**B**) after the CTB.

**Figure 4:**
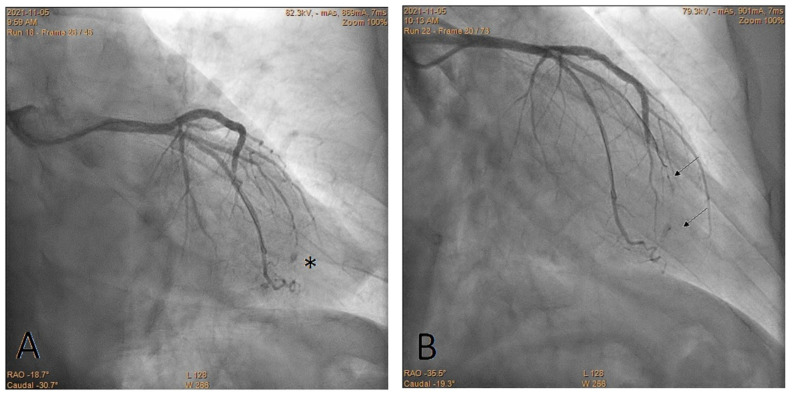
Perforation of the distal obtuse marginal branch. The star shows the location of the perforation. Arrows show markers of the fragmented balloons. (**A**) Before the CTB; (**B**) after the CTB.

**Figure 5:**
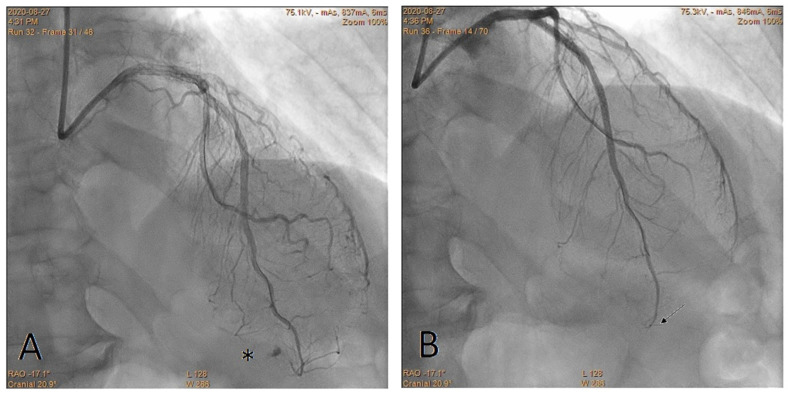
Perforation of the left anterior descending artery. The star shows the location of the perforation. The arrow shows the marker of the fragmented balloon. (**A**) Before the CTB; (**B**) after the CTB.

Other advantages of the CBT include a fixed guidewire position, the opportunity to use several pieces of the cut balloon until the bleeding stops and the utilization of previously used balloons (Figure 3, Figure 4 and Figure 5). Finally, no special or expensive equipment, such as microcatheters and coils, not to mention exceptional operator experience, are required [8].

**Figure 6:**
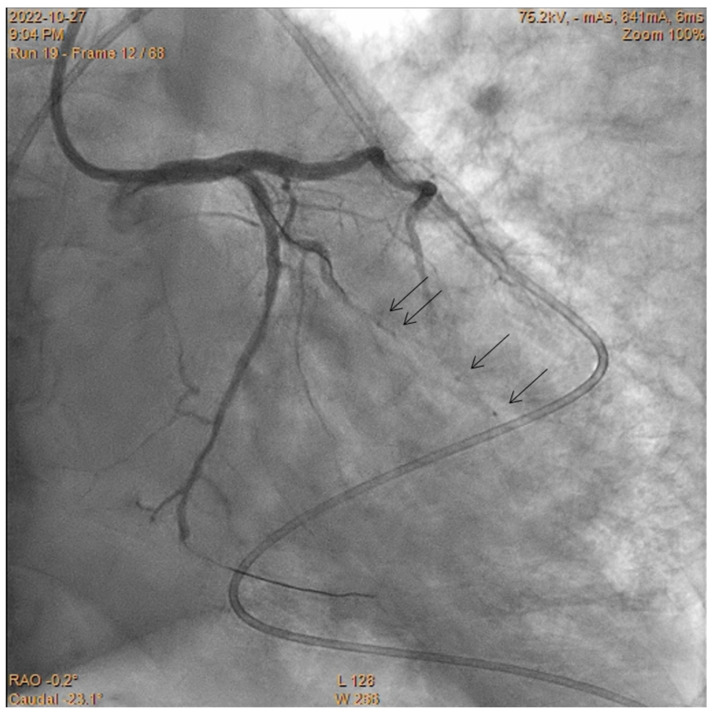
Perforation of the obtuse marginal branch complicated by tamponade requiring pericardiocentesis (catheter in the pericardium). The perforated artery was occluded using CBT. Arrows show markers of the fragmented balloons.

On the contrary, several theoretical disadvantages of the CBT should be discussed. First, the delivery of the cut balloon over the wire can be challenging in the cases of severe calcifications and/or target vessel tortuosity. In such cases, the use of a guide extension or a second guidewire (buddy wire technique) can be advised. Another aspect is the size of the cut balloon, whereby a balloon that is too small might not completely seal the perforation. This situation can occur when the part of balloon is inserted too deep (behind the perforation) or in the case of a mismatch between the size of the balloon and the size of the coronary perforation. Such a scenario can, however, be easily resolved by adding another same-sized or even larger balloon. This was reflected in this study, where in almost half of the cases (48%), embolization with one fragment of the balloon was insufficient, and the addition of a second or more cut balloons was necessary (Figure 6).

Other potential problems of the CBT might include the proximal embolization of the vessel or the inadvertent enlargement of the perforation by using a balloon that is too large. Notably, none of these complications were observed in our patients.

Our study had several limitations. First, it was a relatively small and retrospective study. Nevertheless, to the best of our knowledge, we present the largest cohort of patients with distal coronary perforations treated with the CBT. Second, based on the operator-dependent study inclusion criteria (related to the retrospective nature of the study), we cannot exclude patient selection bias favoring smaller perforations in our cohort. This, however, is difficult to confirm, as all of the examined perforations were deemed clinically relevant and potentially life-threatening at the operators’ discretion. Finally, we did not account for a prospective comparison between the CBT and other embolization techniques.

## 5. Conclusions

The cut balloon technique is feasible, safe, easy to reproduce and highly effective in the embolization of distal coronary perforations encountered during percutaneous interventions. The majority of distal coronary perforations can be sealed with one or two fragments of cut balloons, obviating the need for additional devices and/or sophisticated techniques. Our findings may be considered fairly reproducible given the simplicity and high availability of CBT across most catheterization laboratories.

## Figures and Tables

**Table 1:** Baseline clinical characteristics.

	CBT *n* = 26
Age, years	71 ± 10.6
≥80	7 (27%)
Male	18 (69%)
Acute coronary syndrome	15 (58%)
STEMI	4 (16%)
NSTEMI/UA	11 (42%)
Chronic coronary syndrome	11 (42%)
Previous MI	9 (35%)
Previous PCI	9 (35%)
Previous CABG	0
Diabetes	7 (27%)

CBT—cut balloon technique; STEMI—ST elevation myocardial infarction; NSTEMI—non-ST-elevation myocardial infarction; UA—unstable angina; MI—myocardial infarction; PCI—percutaneous coronary interventions; CABG—coronary artery bypass grafting.

**Table 2:** Angiographic characteristics, management and in-hospital prognosis.

	CBT *n* = 26
PCI type	
CTO	3 (11%)
Non-CTO	23 (89%)
Target vessel for PCI	
Left main coronary artery	2 (8%)
Left anterior descending artery	11 (42%)
Circumflex artery	7 (27%)
Right coronary artery	6 (23%)
ACC/AHA lesion classification	
A	7 (27%)
B1	8 (31%)
B2	8 (31%)
C	3 (11%)
Perforated coronary artery	
Left anterior descending artery	9 (34%)
Diagonal branch	2 (8%)
Intermediate branch	1 (4%)
Obtuse marginal branch	7 (27%)
Right posterolateral artery	4 (15%)
Right posterior descending artery	2 (8%)
Septal collateral	1 (4%)
Device responsible for distal/septal collateral perforation	
Non-hydrophilic guidewire	23 (88%)
Hydrophilic guidewire	2 (8%)
Microcatheter	1 (4%)
Number of guidewires used during procedure	
1	10 (39%)
2	12 (46%)
≥3	4 (15%)
Clinical management	
Pericardiocentesis	4 (16%)
Protamine	0
Prolonged balloon inflation	25 (100%)
Number of balloons used to close the perforation	
1	12 (46%)
2	6 (23%)
3	5 (19%)
4	1 (4%)
5	2 (8%)
In-hospital prognosis	
Cardiac tamponade	4 (16%)
Emergency surgery	0
Death	0

CBT—cut balloon technique; PCI—percutaneous coronary interventions; CTO—chronic total occlusion; ACC/AHA—American College of Cardiology/American Heart Association.

**Table 3:** Advantages and disadvantages of various embolization techniques to treat coronary artery perforation.

Embolization Techniques	Advantages	Disadvantages
Coils	reliable treatment effect	high cost, need for special (delivery and release) equipment, limited operator experience
CBT	high availability, low cost, simplicity, no need for special (delivery) equipment, potentially repeatable if unsuccessful	constrained deliverability
Thrombin	low cost	limited operator experience, need for special (delivery) equipment, limited availability
Fat	high availability, cost-free	limited operator experience, need for special (delivery) equipment, uncertain effects, limited data in the literature
Clot	high availability, cost-free	limited operator experience, need for special (delivery) equipment, uncertain effects, limited data in the literature

## Data Availability

Data is contained within the article.
